# Impact of multi-professional simulation-based training on perceptions of safety and preparedness among health workers caring for coronavirus disease 2019 patients in Pakistan

**DOI:** 10.3352/jeehp.2020.17.19

**Published:** 2020-06-29

**Authors:** Jamal Azfar Khan, Muhammad Rizwan Bashir Kiani

**Affiliations:** 1 Department of Internal Medicine, Combined Military Hospital Landi Kotal Cantt, Khyber Pakhtunkhwa, Pakistan; 2 Army Medical College, Rawalpindi, Pakistan; Hallym University, Korea

**Keywords:** Coronavirus, COVID-19, Pakistan, Severe acute respiratory syndrome coronavirus 2, Simulation training

## Abstract

This study aimed to evaluate perceptions of safety and preparedness among health workers caring for coronavirus disease 2019 (COVID-19) patients before and after a multi-professional simulation-based course in Pakistan. Health workers’ perceptions of preparedness, safety, and their willingness to care for COVID-19 patients were measured before and after they attended a simulation-based training course to prepare them to care for COVID-19 patients at Combined Military Hospital Landi Kotal Cantt, from March 1 to April 30, 2020. The participants’ perceived level of safety and preparedness to care for COVID-19 patients before the simulation-based course was low, but increased after completing it (P<0.05). They felt confident and were significantly more willing to care for patients with COVID-19 or other infections requiring strict isolation. Simulation-based training is an effective tool to improve perceptions of risk and readiness to deal with COVID-19 among medical and non-medical health workers in Pakistan.

## Background/rationale

The first case of coronavirus disease 2019 (COVID-19) in Pakistan was reported in Karachi, on February 26, 2020 [1]. At Combined Military Hospital (CMH) Landi Kotal Cantt in Pakistan, we arranged a special course for COVID-19. We formulated a training course that also included the non-medical staff of the hospital, including those working in the laundry service, security, and sanitation departments. The people in these professions are also at risk of being exposed to droplet-borne infections such as COVID-19. We also included simulation-based exercises in the training course. These exercises included carrying out clinical procedures such as endotracheal intubation, collection of blood samples, and collection of nasopharyngeal and oropharyngeal samples for polymerase chain reaction (PCR) testing for the severe acute respiratory syndrome coronavirus 2 on a manikin. We used mock patients to practice safe transport from one department of the hospital to another by the security staff. We simulated spillages of blood and vomitus on floor and linen for the sanitation and laundry staff to practice dealing with highly infectious materials and surfaces.

## Objectives

This study aimed to determine whether health workers’ perceived levels of preparedness, safety, and willingness to care for COVID-19 patients changed after attending the simulation-based course. We hypothesized that this simulation-based training would impart confidence and a sense of preparedness to care for COVID-19 patients.

## Ethics statement

We obtained permission from the Hospital Ethical Board to conduct our study (02/2020). Informed consent was obtained from the participants.

## Study design

This was a survey-based descriptive study.

## Participants

The participants in the study included nurses, doctors, nursing assistants, sanitation workers, ambulance drivers, and laundry service providers who attended the COVID-19 training course. Hospital staff who would not have any contact with patients or infectious materials (e.g., those on administrative duties) were excluded. Personnel with pre-existing medical conditions such as pulmonary diseases, ischemic heart disease, diabetes mellitus, or who were immunocompromised were also excluded from participation, as they are prone to have complications if they contract COVID-19 [2].

## Setting

The study was conducted at CMH Landi Kotal Cantt, Khyber District from March 1 to April 30, 2020. This hospital is 1 of the 2 hospitals in Landi Kotal that have been designated to care for COVID-19 patients.

The hospital education department formulated the multi-professional training course to put the hospital action plan for COVID-19 into practice. The biosafety and infectious disease protocols of the hospital had already been revised and updated for COVID-19 as per the guidelines of the National Institute of Health in Islamabad. The training course was formulated in consultation with a medical and infectious disease specialist to cover the following aspects of the management of suspected and confirmed COVID-19 patients: (1) personal protective equipment (PPE) use; (2) the hospital’s biosafety protocols; (3) procedures such as oropharyngeal and nasopharyngeal sampling for PCR tests, intravenous blood sampling, patients’ bodily hygiene, and cleaning performed by the clinical staff on the patient; and, (4) procedures such as disinfection of surfaces and cleaning of infectious fluids (e.g., blood and vomitus) by sanitation staff, transport of patients and subsequent disinfection of the ambulance by the ambulance staff, clearing the corridors while maintaining patient isolation by the security staff, and collection and treatment of contaminated linen by laundry managers.

There were interactive classroom sessions and practical sessions in which every participant was required to don and doff PPE including gloves, gowns, goggles, N95 (National Institute for Occupational Safety and Health-approved) and surgical masks, face shields, splash-resistant aprons, and whole-body anti-viral suits. Clinical procedures were practiced using simulation techniques with the help of mock patients and manikins. Multiple scenarios were simulated and practiced as per the hospital protocols.

## Measurement

All the participants of the training course were invited to participate in our study. We obtained permission to use and modify the questionnaire used by Carvalho et al. [3]. The questionnaire consisted of 2 parts. The first part contained questions about participants’ characteristics such as age, sex, their field of work/department, years of experience, any experience of participating in simulation-based training, and experience of caring for patients with infectious diseases. The second part contained 8 questions regarding their perceptions of risk and their preparedness to work with patients with COVID-19 or other infections requiring strict isolation (biosafety level III/ IV) (Supplement 1). The participants responded on a 10-point Likert scale. They were asked to complete the questionnaire before starting the training course and after finishing it.

## Statistical analysis

The data were analyzed using IBM SPSS Statistics ver. 23.0 (IBM Corp., Armonk, NY, USA). The frequency and percentages of categorical variables were calculated. For continuous variables, the median and interquartile range (25th–75th percentiles) were calculated. The Mann-Whitney U-test was used to test the significance of differences between independent samples.

## Participant characteristics

The demographic characteristics of the 44 participants are shown in Table 1.

## Descriptive data

**Fig. 1.**
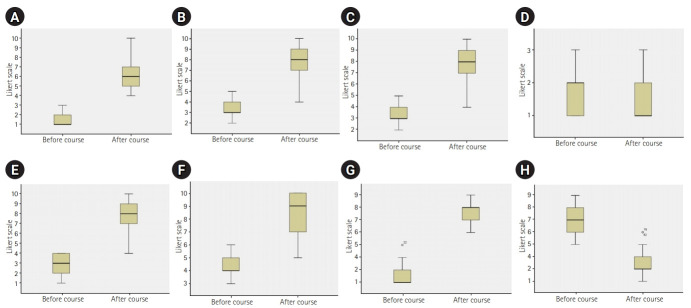
Answers to the questions regarding perceptions of preparedness, risk, and willingness to care for patients with COVID-19 or other infections requiring high isolation before and after the simulation-based training course. (A) Q1: I feel ready to participate in the management of patients infected with COVID-19. (B) Q2: I feel ready to participate in the management of patients with infections that require high isolation. (C) Q3: I think that my colleagues who are participating in the course are ready to manage patients with COVID-19 or other infections that require high isolation. (D) Q4: I think that my colleagues who are not participating in the course are ready to manage patients with COVID-19 or other infections that require high isolation. (E) Q5: I feel safe if I had to participate in the management of a patient infected with COVID-19. (F) Q6: If today I have to take care of a patient infected with COVID-19, I would do it. (G) Q7: If today I have to take care of a patient with an infection requiring high isolation measures, I would do it. (H) Q8: I am afraid to participate in the management of a patient infected with COVID-19 or other agents requiring high isolation. COVID-19, coronavirus disease 2019.

After the course, all the questionnaire results showed significant changes (P<0.05) (Dataset 1). The participants felt better prepared to take care of patients with infections requiring strict isolation such as COVID-19. They considered other participants of the course to be ready for it too. Moreover, their perceptions regarding the preparedness of those colleagues who had not attended the course were significantly lower. They felt safer and were significantly more willing to participate in the management of COVID-19 patients. Finally, their fear of working with a patient requiring high isolation was also reduced after attending the course. The responses provided by the participants before and after the course are shown in Fig. 1 using box plots.

## Interpretation

Our results showed that at the start of the course, the participants perceived themselves to be neither prepared nor motivated to take care of COVID-19 patients. They also did not feel safe in dealing with such patients. However, at the end of the course, there were significant improvements in perceptions of preparedness, willingness to work, and the feeling of being safe when working with patients with COVID-19. Varying results have been reported in other studies regarding perceptions of preparedness after simulation-based training. Prescott and Garside [4] reported that all the participants of their study felt better prepared for the assigned tasks after a simulation-based training. However, it has also been reported that fewer participants felt prepared for the tasks for which they had received simulation-based training [5]. Our study showed a significant improvement in perceptions of preparedness among the participants after the training.

The participants in our training course believed from the beginning that their colleagues who were not attending the course were not prepared to handle COVID-19 patients. At the end of the course, this perception was strengthened. This implies that they perceived that the course had prepared them for the challenge of caring for COVID-19 patients as compared to their colleagues who had not trained with them. We interpreted this result as validating the effectiveness of our new multi-disciplinary training course.

We saw that the feeling of fear regarding caring for infectious patients requiring strict isolation was reduced after the participants had completed the course. Fear is an irrational feeling that can hamper performance. Fear can also hamper learning. It is important that health personnel feel safe while learning, and simulation-based training provides a safe environment to learn to deal with a broad range of potentially dangerous situations [6]. Thus, simulation-based learning effectively imparts confidence, competence, and knowledge to the learner. If they feel safe, we can assume that they would be able to deliver better care to the patients.

At the end of simulation-based training, there is usually a debriefing session, which is considered to be a crucial part of the learning exercise. We also found debriefing to be invaluable in our course. It was used to reinforce good practices and to reflect upon, discuss, and improve aspects that had not been performed up to the mark. It gave the participants insights and helped them develop a better understanding of the rationale behind the procedures.

It has been reported that repeated team-based simulation training increased the frequency of task completion [7]. The reason may be either familiarity with the role that each member of the team plays in a given situation or reduction in anxiety with practice. Thus, we recommend that essential skills and procedures should be practiced through regular simulation-based training programs organized at hospitals.

## Limitations

The sample size was small and the study was conducted at only 1 hospital. Therefore, the results cannot be generalized. It is recommended that we follow up this study with a larger sample size and conduct it at multiple institutions. Furthermore, simulations should be compared with other training methods to clarify their effect on perceptions of safety and preparedness to care for patients with infections requiring strict isolation. This will help in formulating appropriate training programs for health care workers from different departments and will yield maximum benefits.

## Conclusion

Multi-professional simulation-based training imparted confidence and a sense of preparedness among health workers to care for patients with infections that require strict isolation, such as COVID-19. The feelings of insecurity and fear regarding caring for such patients were reduced through such training programs.

## Figures and Tables

**Table 1.** Characteristics of the participants in the COVID-19 training course

Participant characteristic	No (%)
Age (yr)	
21–30	26 (59)
31–40	14 (31.8)
41–50	4 (10)
Sex	
Male	36 (81.8)
Female	8 (18.2)
Professional experience (yr)	
≤5	7 (15.9)
6–10	23 (52.3)
11–15	11 (25)
16–20	3 (6.8)
Specialty	
Nursing assistants	19 (43.1)
Sanitation workers	8 (18.2)
Doctors	7 (15.9)
Security staff	4 (10)
Nurses	3 (6.8)
Laundry staff	3 (6.8)
Experience with simulation	
Yes	5 (11.4)
No	39 (88.6)
Experience of working with infectious patients requiring isolation	
Yes	18 (40.9)
No	26 (59.1)

COVID-19, coronavirus disease 2019.

## Footnotes

**Data availability** Data files are available from Harvard Dataverse: https://doi.org/10.7910/DVN/Z3KGW8 Dataset 1. Raw data.

## Supplementary materials

Data files are available from Harvard Dataverse: https://doi.org/10.7910/DVN/Z3KGW8

Supplement 1. Questionnaire assessing the perceived level of preparedness and safety regarding the management of infected patients that require high isolation.

Supplement 2. Audio recording of the abstract.
